# Genetic anticipation in Swedish Lynch syndrome families

**DOI:** 10.1371/journal.pgen.1007012

**Published:** 2017-10-31

**Authors:** Jenny von Salomé, Philip S. Boonstra, Masoud Karimi, Gustav Silander, Marie Stenmark-Askmalm, Samuel Gebre-Medhin, Christos Aravidis, Mef Nilbert, Annika Lindblom, Kristina Lagerstedt-Robinson

**Affiliations:** 1 Department of Molecular Medicine and Surgery, Karolinska Institutet, and Department of Clinical Genetics, Karolinska University Hospital, Solna, Stockholm, Sweden; 2 Department of Biostatistics, University of Michigan, Ann Arbor, Michigan, United States of America; 3 Department of Oncology, Radiumhemmet, Karolinska University Hospital, Solna, Stockholm, Sweden; 4 Department of Radiation Sciences, Umeå University, Umeå, Sweden; 5 Department of Oncology, Linköping University, Linköping, Sweden; 6 Department of Clinical Genetics, Office for Medical Services, Division of Laboratory Medicine, Lund, Sweden; 7 Division of Clinical Genetics, Department of Laboratory Medicine, Lund University, Lund, Sweden; 8 Department of Immunology, Genetics and Pathology, Uppsala University, Uppsala, Sweden; 9 Department of Clinical Sciences, Division of Oncology and Pathology, Lund University, Lund, Sweden; 10 Clinical Research Centre, Hvidovre Hospital, Copenhagen University, Hvidovre, Denmark; Section of Medical Genetics, ITALY

## Abstract

Among hereditary colorectal cancer predisposing syndromes, Lynch syndrome (LS) caused by mutations in DNA mismatch repair genes *MLH1*, *MSH2*, *MSH6* or *PMS2* is the most common. Patients with LS have an increased risk of early onset colon and endometrial cancer, but also other tumors that generally have an earlier onset compared to the general population. However, age at first primary cancer varies within families and genetic anticipation, i.e. decreasing age at onset in successive generations, has been suggested in LS. Anticipation is a well-known phenomenon in e.g neurodegenerative diseases and several reports have studied anticipation in heritable cancer. The purpose of this study is to determine whether anticipation can be shown in a nationwide cohort of Swedish LS families referred to the regional departments of clinical genetics in Lund, Stockholm, Linköping, Uppsala and Umeå between the years 1990–2013. We analyzed a homogenous group of mutation carriers, utilizing information from both affected and non-affected family members. In total, 239 families with a mismatch repair gene mutation (96 *MLH1* families, 90 *MSH2* families including one family with an *EPCAM–MSH2* deletion, 39 *MSH6* families, 12 *PMS2* families, and 2 *MLH1+PMS2* families) comprising 1028 at-risk carriers were identified among the Swedish LS families, of which 1003 mutation carriers had available follow-up information and could be included in the study. Using a normal random effects model (NREM) we estimate a 2.1 year decrease in age of diagnosis per generation. An alternative analysis using a mixed-effects Cox proportional hazards model (COX-R) estimates a hazard ratio of exp(0.171), or about 1.19, for age of diagnosis between consecutive generations. LS-associated gene-specific anticipation effects are evident for *MSH2* (2.6 years/generation for NREM and hazard ratio of 1.33 for COX-R) and *PMS2* (7.3 years/generation and hazard ratio of 1.86). The estimated anticipation effects for *MLH1* and *MSH6* are smaller.

## Author summary

Genetic anticipation is a phenomenon where symptoms of a hereditary disease appear at an earlier age and/or are more severe in successive generations. In genetic disorders such as Fragile X syndrome, Myotonic dystrophy type 1 and Huntington disease, anticipation is caused by the expansion of unstable trinucleotide repeats during meiosis. Anticipation is also reported to occur in some hereditary cancers though the underlying mechanism behind this observation is unknown. Several studies have investigated anticipation in Lynch syndrome, the most common hereditary colorectal cancer syndrome, yet there is a debate concerning whether anticipation occurs and what underlying mechanism there is. The objective of this project is to study if anticipation is part of the clinical picture in Swedish families with LS, with the long term goal to enable better prediction of age at onset in family members. Our results suggest that anticipation occurs in families with mutation in *MSH2* and *PMS2*, while the evidence is equivocal for *MLH1* and *MSH6*.

## Introduction

Lynch syndrome (LS) is an autosomal dominant inherited syndrome that increases the risk of cancer, primarily in the colon, the rectum and the endometrial lining of the uterus, and to a lesser degree also in the stomach, the ovary, the hepatobiliary tract, the urinary tract, the small bowel and the brain [1,2]. LS is one of the most common heritable cancer syndromes, accounting for up to 4% of the total colorectal cancer burden in Europe, where patients have up to 70% lifetime risk of developing colorectal or endometrial cancer at an early age [1]. LS was formerly known as hereditary non-polyposis colorectal cancer (HNPCC), but when clinical criteria evolved to take into account not only colorectal cancer to identify families with LS [3,4] the name Lynch Syndrome became generally accepted [5]. Today the diagnosis LS is restricted to families with a known pathogenic germline mutation in one of the mismatch repair (MMR) genes *MLH1*, *MSH2*, *MSH6* and *PMS2* irrespective of family history [6,7]. The MMR system corrects indels or mismatches in the DNA, and is evolutionary conserved from bacteria to human [8]. In human the recognition of nucleotide mismatches is mediated by the protein heterodimers *MSH2/MSH6* or *MSH2/MSH3*, while the removal and resynthesis of nucleotides is mediated by *MLH1/PMS2* [9].

LS is a heterogeneous disease with regard to tumor spectrum and age at onset [10]. Part of this phenotypic variation has been linked to specific MMR gene mutation. For instance, *MLH1* mutation carriers are suggested to have a higher risk for colorectal cancer (CRC) and earlier age of onset, compared to *MSH2* and *MSH6* mutation carriers [11–15]. In general, *MSH6* mutation carriers tend to have a later age of onset and lower penetrance for LS associated tumors, apart from endometrial cancer, compared to *MLH1* and *MSH2* mutation carriers [16–20]. An older age of onset and a lower overall risk for CRC has also been suggested for *PMS2* mutation carriers [21,22]. However, LS display phenotypic variation in age of onset also within families and between families with the same mutation [23–25]. This variation is attributed to individual genetic differences modifying the effect of an inherited MMR mutation [26–31]. Another factor proposed to influence age at onset is genetic anticipation, defined as progressive earlier onset and severity of disease in successive generations within a family. This phenomenon is closely related to the disease mechanisms in several genetic disorders, e.g the neurodegenerative diseases Fragile X syndrome, Myotonic dystrophy type 1 and Huntington disease, in which trinucleotide repeat expansion directly influence expressivity and penetrance of disease [32]. Anticipation has also been observed in hereditary cancer for example familial melanoma, Li-Fraumeni syndrome, breast, ovarian and pancreatic cancer, and recently in the renal cell cancer syndromes von Hippel-Lindau and HLRCC (hereditary leiomyomatosis and renal cell cancer) [33–39]. In LS, a progressive decrease of age at CRC onset was proposed already in 1925 when the syndrome was first described [40,41]. However, it is complicated to estimate genetic anticipation and there are contradictory reports regarding its existence in LS, though the majority indicate anticipation [42–52]. Previous studies have applied various statistical methodologies, compiled different mutations and included subjects with LS associated mutations as well as subjects with only a clinical diagnosis. In light of these studies, we analyzed affected and unaffected mutation carriers in LS families throughout Sweden, to investigate signs of anticipation using two regression models with adjustment for potential confounders, including gene-specific effects.

## Materials and methods

In Sweden, families with suspected LS are referred to the regional department of Clinical genetics in Umeå, Uppsala, Stockholm, Linköping, Göteborg or Lund, for counceling and genetic testing. Out of this population-based cohort, families identified with a LS-associated MMR mutation that received genetic counseling in Lund, Stockholm, Linköping, Uppsala or Umeå between January 1990 and December 2013 were enrolled in this study. This project was approved in accordance with the Swedish legislation of ethical permission (2003:469). All patients provided oral or written informed consent for genetic diagnostics as part of their routine clinical care. This anonymized genetic information may be used for research without further consent sought from the patients if approved by an ethical review board. Accordingly, this study was approved by the Regional ethical review board in Stockholm (dnr 2014/1320-31).

Patient and family cancer history was reported at the time of genetic counseling and cancer diagnoses were further confirmed from medical records or pathology reports. A total of 239 families with proven pathogenic MMR variants described in [53] (96 *MLH1* families, 90 *MSH2* families including one *EPCAM-*deletion family, 39 *MSH6* families, 12 *PMS2* families, and 2 *MLH1+PMS2* families), comprising 1029 mutation carriers, were identified in the cohort. One individual whose parents were both mutation carriers was excluded. Additionally, the sex of 11 carriers was unknown, and the age at diagnosis for an additional 14 was missing. We excluded these 25 individuals, leaving 1003 at-risk carriers with available follow-up information and sufficient pathological and medical information to be included in the study. We grouped the *EPCAM-*deletion family within the *MSH2* families, as it is reported that a partly deleted *EPCAM* gene (located upstream of *MSH2*) cause LS through reducing the expression of *MSH2* in *EPCAM*-expressing tissues [54]. For statistical modeling purposes, we counted two families with mutations in both *MLH1* and *PMS2* as having mutations in *PMS2* only (unreported auxiliary analyses that excluded these families altogether or counted them as *MLH1* showed that our findings are not sensitive to this decision). The follow-up period was defined as the time from birth until age at onset, and for individuals who were diagnosed with multiple Lynch-related cancers, age of onset was recorded as the time of first diagnosis. Our first analytic approach was the normal random effects model (NREM) proposed by Larsen et al. [45], which has been used previously to test for anticipation in LS [43] and *BRCA*-mutation related cancers [55]. Let *n*_*i*_ denote the number of carriers in the *i*th family, *i = 1*, *2*, *…*, *239*, and let *j = 1*, *2*, *…*, *n*_*i*_ index the *j*th individual in family *i*. The NREM is given by

$$T_{ij}=\mu_{i}+\gamma Z_{ij}+\beta X_{ij}+\varepsilon_{ij},$$ (1)

where *T*_*ij*_ is the age of diagnosis in years for the *j*th member of family *i* (“person *ij*”), *μ*_*i*_ is the family-specific random intercept representing a typical age of diagnosis in the *i*th family, *Z*_*ij*_ is person *ij*’s generation (coded with respect to oldest observed generation in each family, as described in [41] and *γ*, the parameter of interest, is the mean change in age of diagnosis between consecutive generations, i.e. the anticipation effect. In the NREM, anticipation is indicated if *γ < 0*. Collectively the *X*_*ij*_ term represents any other covariate(s) of interest for person *ij*, the effect of which is given by *β*. The final term *ε*_*ij*_ is the residual error, assumed to be independently and normally distributed with mean zero and variance *σ*^*2*^. For each person who was not diagnosed with a Lynch-associated cancer during the follow-up period, the likelihood contribution is given by the normal survivor function, that is, the probability of being cancer-free at the age of last follow-up. We assume that the censoring mechanism is independent of the time to cancer diagnosis.

Our second analytic approach, which is also a regression strategy, extends the Cox proportional hazards model that was used in [56] to test for anticipation in lymphoproliferative tumors. Person *ij*’s hazard for cancer diagnosis at age *t* is modeled as:

$$\lambda (t|Z_{ij},X_{ij})=\lambda_{0}(t)\exp({\tilde{\mu }}_{i}+\tilde{\gamma }Z_{ij}+\tilde{\beta }X_{ij}).$$ (2)

The function *λ*_*0*_*(t)* is the overall baseline hazard function. In Daugherty et al., the baseline hazard was assumed to be identical for all families, that is, ${\tilde{\mu }}_{i}$ was not included in the model and within-family correlations were accounted for by robust sandwich-type covariance estimates. Here we add a random family-level effect ${\tilde{\mu }}_{i}$, similar to NREM, which makes the less restrictive assumption that all families’ baseline hazards are proportional to, rather than equal to, one another. We call this Cox model with family-level random effect COX-R. The remaining parameters are analogous to NREM. Specifically, $\tilde{\gamma }$ gives the generational effect of anticipation as a log-hazard ratio, with $\tilde{\gamma }>0$ indicating anticipation, and $\tilde{\beta }$ is the log-hazard ratio(s) for all other covariates.

In addition to adjusting for sex, we also included mutational status in NREM and COX-R. In Eqs (1) and (2), let person *ij*’s length-4 vector of covariates be given by *X*_*ij*_ *= {1[sex*_*ij*_ *=* male], *1*[*gene*_*i*_ *= MSH2]*, *1*[*gene*_*i*_ *= MSH6]*, *1*[*gene*_*i*_ *= PMS2]}*, where *1[y]* is the indicator function, equal to 1 if *y* is true, sex_ij_ is the sex of person *ij* and *gene*_i_ is the mutational status of family *i*. *MLH1* serves as the reference category.

We also investigated whether there were gene-specific effects of anticipation by substituting *Z*_*ij*_ in Eqs (1) and (2) with the four dimensional covariate vector.

$$\begin{array}{c} Z_{ij}^{*}=Z_{ij}\times \{1[{gene}_{i}=MLH1],1[{gene}_{i}=MSH2],\\ 1[{gene}_{i}=MSH6],1[{gene}_{i}=PMS2]\}. \end{array}$$

All analysis was done in the R software package R Core Team [57]. Code for maximizing the integrated partial likelihood of model (2), marginalized over the random effects ${\tilde{\mu }}_{i}$, is provided in the R package COXME [58].

## Results

**Fig 1:**
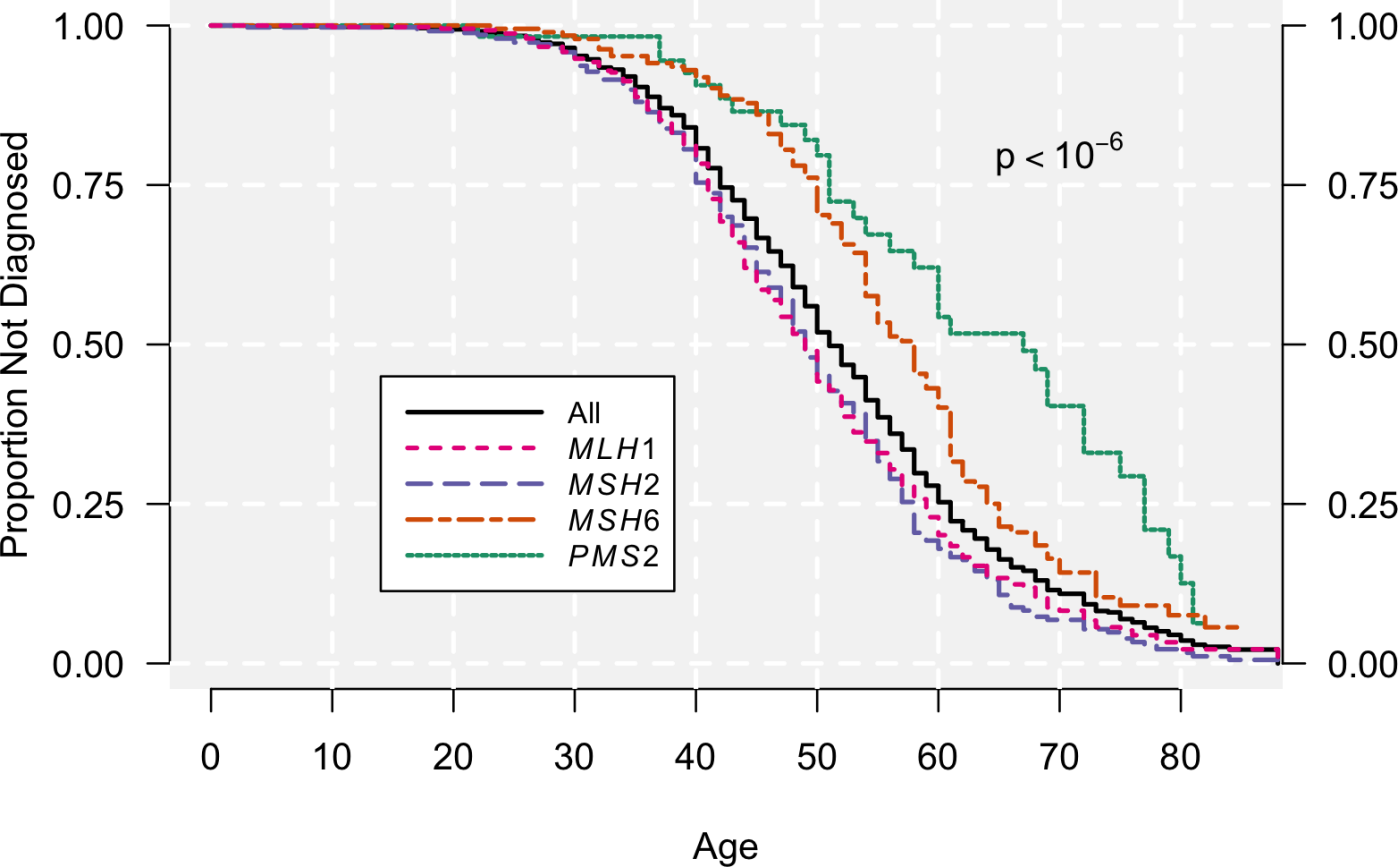
Kaplan-Meier estimates of time to first Lynch-associated cancer diagnosis in the Swedish Registry data, both overall and stratified by mutational status. The x axis displays age (years) and the y axis displays the probability of being free from diagnosis.

Table 1 presents the clinical characteristics of our data and Fig 1 plots the Kaplan-Meier estimate of the time to first Lynch-associated cancer diagnosis, to give an overview of the age at onset in our cohort. During the follow-up period, 719 carriers were diagnosed with at least one Lynch-associated cancer and 171 were diagnosed with multiple Lynch-associated cancers. Overall, the median age of first diagnosis was 51 years (95% CI: 50–53), but this varied with mutational status, being 49 years in both *MLH1* and *MSH2* patients and 58 and 67 years, respectively, for *MSH6* and *PMS2* patients.

**Table 1:** Clinical characteristics of the analyzed LS cohort.

	All	*MLH1*	*MSH2*	*MSH6*	*PMS2*^*a*^
Family-level characteristics					
No. families	239	96	90	39	14
Mean No.	4.2	4.2	3.8	4.9	4.1
carriers/fam					
Mean No. total diagnoses/fam	3.0	3.0	3.0	3.3	2.4
Mean No. generations/fam	2.5	2.6	2.5	2.6	1.9
Range No. generations/fam	(1–5)	(1–4)	(1–5)	(1–4)	(1–3)
Individual-level characteristics					
No. carriers	1003	407	345	193	58
Median age of first diagnosis (95% CI)	51	49	49	58	67
	(50–53)	(47–51)	(48–51)	(55–60)	(58–75)
Median follow-up	72	66	Not reached	69	71
% female	52.2	48.6	51.0	65.2	41.4

^*a*^Two families with germline mutations in both *MLH1* and *PMS2* are included in the *PMS2* category.

Based on this, in addition to adjusting for sex, we also included mutational status in NREM and COX-R analyses. Table 2 gives the estimates, standard errors, and Wald-type *p*-values for the anticipation effects only, and Table 3 provides all parameter estimates. As shown in Table 2, the estimates of *γ* (NREM) and $\tilde{\gamma }$ (COX-R) are -2.1 (*p* = 0.0001) and 0.171 (*p* = 0.0013), respectively. Both suggest the presence of anticipation: a 2.1 year decrease in the age of diagnosis per generation and a hazard ratio of exp(0.171) = 1.19 between consecutive generations. Because mutational status appears to confound the age of diagnosis, we also investigated whether there were gene-specific effects of anticipation, yielding one estimated anticipation effect for each analyzed MMR-gene. These are given in the bottom rows of Table 2. In NREM, anticipation is estimated to be -1.8 (*p* = 0.044), -2.6 (*p* = 0.003), -1.1 (*p* = 0.366), and -7.3 (*p* = 0.014) years per generation respectively, for *MLH1*, *MSH2*, *MSH6*, and *PMS2*. In COX-R, the corresponding log-hazard ratios are 0.127 (*p* = 0.133), 0.284 (*p* = 0.001), -0.005 (*p* = 0.965), and 0.618 (*p* = 0.052), representing hazard ratios of 1.13, 1.33, 0.99, and 1.86, respectively. In both models, the confidence intervals (CIs) for the anticipation effects of *MSH2* and *PMS2* lie far from their null values (Table 2), whereas there is greater uncertainty with regard to any possible effect of anticipation in *MLH1* and *MSH6*.

**Table 2:** Results corresponding to the anticipation effects for the NREM and COX-R models, without and with the gene-generation interactions (Est = Estimate, CI = 95% Confidence Interval).

	NREM			COX-R		
Model	Parameter	Est. (CI)	Wald *p*-val	Parameter	Est. (CI)	Wald *p*-val
No. Interactions	*γ*	-2.10	0.0001	$\tilde{\gamma }$	0.171	0.001
		(-3.16,-1.03)			(0.066,0.275)	
Gene-Generation Interactions	*γ*_*MLH1*_	-1.76	0.044	${\tilde{\gamma }}_{\mathrm{MLH}1}$	0.127	0.133
		(-3.46,-0.05)			(-0.038,0.292)	
	*γ*_*MSH2*_	-2.55	0.003	${\tilde{\gamma }}_{\mathrm{MSH}2}$	0.284	0.001
		(-4.22,-0.87)			(0.123,0.446)	
	*γ*_*MSH6*_	-1.10	0.366	${\tilde{\gamma }}_{\mathrm{MSH}6}$	-0.005	0.965
		(-3.48,1.28)			(-0.236,0.225)	
	*γ*_*PMS2*_	-7.33	0.014	${\tilde{\gamma }}_{\mathrm{PMS}2}$	0.618	0.052
		(-13.2,-1.47)			(-0.004,1.241)	

**Table 3:** Complete results for the NREM and COX-R models, without and with the gene-generation interactions (Est = Estimate, CI = 95% Confidence Interval; SE = standard error).

	NREM			COX-R		
Model	Parameter	Est. (CI)	Wald *p*-val	Parameter	Est. (CI)	Wald *p*-val
No interactions	*E(μ*_*i*_*)*	53.2	<0.0001			
		(50.6,55.8)				
	*γ*	-2.10	0.0001	$\tilde{\gamma }$	0.171	0.001
		(-3.16,-1.03)			(0.066,0.275)	
	*β*_*sex*_	0.82	0.352	${\tilde{\beta }}_{\mathrm{sex}}$	-0.121	0.138
					(-0.280,0.039)	
		(-0.91,2.55)				
	*β*_*MSH2*_	-0.08	0.945	${\tilde{\beta }}_{\mathrm{MSH}2}$	0.003	0.980
		(-2.23,2.08)				
					(-0.197,0.202)	
	*β*_*MSH6*_	7.83	<0.0001	${\tilde{\beta }}_{\mathrm{MSH}6}$	-0.655	<0.0001
		(5.14,10.5)			(-0.910,-0.400)	
	*β*_*PMS2*_	11.35	<0.0001	${\tilde{\beta }}_{\mathrm{PMS}2}$	-0.939	<0.0001
					(-1.374,-0.504)	
		(6.89,15.8)				
	*Var(μ*_*i*_*)*	9.53		$\mathrm{Var}({\tilde{\mu }}_{i})$	0.101	
		(SE = 4.66)				
	*Var(ε*_*ij*_*)*	151.5				
		(SE = 8.96)				
Gene-Generation Interactions	*E(μ*_*i*_*)*	52.2 (1.8)	<0.0001			
	*γ*_*MLH1*_	-1.76	0.044	${\tilde{\gamma }}_{\mathrm{MLH}1}$	0.127	0.133
		(-3.46,-0.05)			(-0.038,0.292)	
	*γ*_*MSH2*_	-2.55	0.003	${\tilde{\gamma }}_{\mathrm{MSH}2}$	0.284	0.001
		(-4.22,-0.87)			(0.123,0.446)	
	*γ*_*MSH6*_	-1.10	0.366	${\tilde{\gamma }}_{\mathrm{MSH}6}$	-0.005	0.965
		(-3.48,1.28)			(-0.236,0.225)	
	*γ*_*PMS2*_	-7.33	0.014	${\tilde{\gamma }}_{\mathrm{PMS}2}$	0.618	0.052
		(-13.2,-1.47)			(-0.004,1.241)	
	*β*_*sex*_	0.87	0.328	${\tilde{\beta }}_{\mathrm{sex}}$	-0.125	0.126
					(-0.285,0.035)	
		(-0.85,2.59)				
	*β*_*MSH2*_	1.39	0.495	${\tilde{\beta }}_{\mathrm{MSH}2}$	-0.270	0.231
					(-0.712,0.172)	
		(-3.49,6.28)				
	*β*_*MSH6*_	6.53	0.029	${\tilde{\beta }}_{\mathrm{MSH}6}$	-0.415	0.146
					(-0.974,0.145)	
		(0.39,12.7)				
	*β*_*PMS2*_	19.4	<0.0001	${\tilde{\beta }}_{\mathrm{PMS}2}$	-1.61	0.002
					(-2.62,-0.608)	
		(9.41,29.3)				
	*Var(μ*_*i*_*)*	9.81		$\mathrm{Var}({\tilde{\mu }}_{i})$	0.109	
		(SE = 4.76)				
	*Var(ε*_*ij*_*)*	150.4				
		(SE = 8.94)				

## Discussion

We investigated signs of anticipation in LS through the analysis of a large, Swedish population-based cohort and regression analyses suggest that anticipation exists in these families. The NREM analysis suggests that the age of diagnosis in families is decreasing by about 2 years per generation, and the COX-R analysis suggests a multiplicative increase in the rate of diagnosis of about 1.19 between generations. These regression analyses carry at least two important advantages over hypothesis testing approaches that compare the age of diagnosis between all parent-child pairs. First, they make use of the partial follow-up time from all at-risk carriers who have not yet been diagnosed; these individuals would otherwise be excluded from analysis. Second, the model-based structure allows for straightforward incorporation of genetic effects or other possible confounders. The underlying causal mutation evidently plays a role in the extent of anticipation, as our estimates varied between MMR genes. Among the MMR genes, the ordering of estimated anticipation effects was *PMS2*, *MSH2*, *MLH1*, and *MSH6*, with the largest effect in *PMS2* (7.3 years/generation [NREM] or a hazard ratio of exp(0.618) = 1.86 [COX-R]). Although the small number of *PMS2* families yielded correspondingly large uncertainty, these effects were still highly significant therefore this uncertainty does not invalidate the findings. For *MSH2*, the estimated effect of anticipation was 2.6 years/generation or a hazard ratio of exp(0.284) = 1.33.

The results are comparable to those reported in several earlier studies (for a review, see [41]). In an analysis of Lynch families from the Danish HNPCC Registry [45], an anticipation effect of about three years/generation was reported but no differences between mutational status was found. A version of the same data was considered again in Boonstra, et al. [43], who fit variants of both regression models considered here, reporting effects of 3.3 years/generation and hazard ratios of exp(0.22) = 1.25. Neither model in that study adjusted for mutational status. Also, the Cox model did not include family-level random effects, as we do here; our approach is arguably a more accurate, although still simplified, reproduction of the true underlying hazard process. A later report analyzed the same Danish HNPCC Registry data with Bayesian modeling techniques [59], allowing anticipation to be random between families. They estimated population-level gene-specific effects of anticipation, as performed here, for *MLH1*, *MSH2*, and *MSH6*. They found respective anticipation effects of 2.8, 2.5, and 1.0 years, consistent with our findings. Several other studies based on anecdotal observation or analyses of affected parent-child pairs have found effects of anticipation varying between 5.5 and 10 years [44,47,49,50]. A notable exception from previous studies is Tsai, et al. [46] who found no evidence for anticipation in 475 parent-child pairs from the Johns Hopkins Hereditary Colorectal Cancer Registry; in part this may be explained by differences in eligibility as only 14 of the 475 parent-child pairs analyzed had verified disease-predispoing germline MMR gene mutations.

The underlying mechanism for anticipation in heritable cancer is still unknown. However, it has been proposed that anticipation is caused by a progressive accumulation of germline mutations, due to the reduced DNA mismatch repair ability in mutation carriers [51]. Accordingly, haploid/monoallelic mutations in the MMR system affect the mutation load in the carrier prior to loss of the second allele, and accumulated alterations in the germ cells is transferred to the offspring [41]. Interestingly, there is an overrepresentation of mononucleotide repeats within and around the human MMR genes compared to other genomic regions, with an overrepresentation in the *PMS2* gene [60,61]. It has been suggested that MMR proteins maintain the length of such microsatellites present within their own nucleotide sequences by an evolutionary mechanism operating by gene-protein interactions [60]. With the above arguments a deficient MMR system would propagate errors through generations and this would be most significant for mutations in the *PMS2* gene, which is in accordance with our results. In addition, it has been shown that *PMS2*-deficient mice eggs forms embryos with an increased mononucleotide mutation rate, indicating that MMR mutations might affect germline mutation rate in a heterozygous state [62]. This also points to our results that *PMS2* mutations carriers would display the most anticipation if the mutation load is inherited by the next generation.

Noteworthy, *PMS2* and *MSH2* are not part of the same protein complex involved in recognition, excision and resynthesis of mismatched nucleotides [63], nor does the *MSH2* gene contain the same magnitude of mononucleotide repeats as *PMS2* [60]. This together suggests a different underlying mechanism generating anticipation in *MSH2* mutation carriers. For example, it is shown that MMR deficiency affect telomere shortening in human fibroblasts, and that this might influence heterozygous carriers of a *MSH2* mutation in particular [64]. Moreover, in a recent study telomere shortening correlated significantly with age at onset in the *MSH2* carriers, whereas the *MLH1* carriers displayed longer telomeres and delayed age at onset [65]. Nevertheless, MMR mutation carriers with LS-associated cancer may have specific telomere-length dynamics but telomere shortening does not alone explain anticipation, as reported by Segui et al [66], indicating that gene-specific dynamics and different mechanisms are involved.

Despite a general concurrence with earlier studies, several caveats accompany our findings. Evidently, our study and previously published evidence that performed survival analysis for genetic anticipation in LS suggests that if genetic anticipation does exist, the effect is modest [42,43,45]. This makes anticipation a difficult problem statistically and challenges some of the clinical utility of our findings. At the population level, anticipation may well also be due to reasons other than genetic. For example, cohort effects arising from changes in treatment, diagnostic or environmental factors can also result in a decline in age at diagnosis. These effects should be visible both within family trees and in the entire population (which is a mix of mutation carriers from different generations). This is in contrast to genetic anticipation, which would only be seen within each unique family tree.

Voskuil, et al. found that the hazard ratio corresponding to generation decreased considerably in magnitude after adjusting for birth cohort [42], although their final estimated hazard ratios for the effect of generation were still very close to our estimate of 1.2. Statistically, birth cohort and generation are typically highly correlated, which can cause the resulting parameter estimates to be unstable. Boonstra, et al. [59] attempted to disentangle these effects by independently estimating secular trends in age of colorectal cancer diagnosis from a cancer registry of all colorectal cancers, and adjusting the Danish HNPCC Registry data for this estimated trend before analysis. Still, the results indicated as reported earlier in this section, population-level gene-specific effects of anticipation of 2.8, 2.5, and 1.0 years, respectively, for *MLH1*, *MSH2*, and *MSH6*. Our estimated effects of anticipation decrease by about 0.7 years when we directly apply the secular trends estimated in Boonstra, et al. [54].

Furthermore, it has been argued that anticipation may be falsely detected due to fecundity bias [48]. Through repeated simulations of parent-child pairs in which no anticipation exists (in truth) but the fertility rate was positively correlated with age of diagnosis, Stupart, et al. demonstrated in a particular scenario that an apparent anticipation effect of about 1.8 years can manifest. Noteworthy, the greatest reduction in fertility was predominantly among those diagnosed before age 29, affecting the fertility of the cohort as a whole. In our cohort, the Kaplan-Meier estimated proportion of patients free of diagnosis at age 29 was 96.5%, which suggests that fecundity bias due to these patients is likely to be small.

Taken together, our findings are in line with those of previous studies. That being said, the study of genetic anticipation is both complex and statistically challenging. The ideal setting in the continuing assessment of fine variations in LS phenotype, such as anticipation, would be prospective, population-based datasets, together with state-of-the-art statistical methods. Still, a number of promising findings have been reported previously, yet often the statistical methods or small sample sizes have been limiting. We believe that the analyses performed in our study properly consider familial, genetic, and clinical parameters and therefore give a representative measurement of anticipation in Lynch Syndrome.
